# Cultural change beyond adoption dynamics: Evolutionary approaches to the discontinuation of contraception

**DOI:** 10.1017/ehs.2021.8

**Published:** 2021-02-09

**Authors:** Alexandra Alvergne, Rose Stevens

**Affiliations:** 1 ISEM, Université de Montpellier, CNRS, IRD, EPHE, Montpellier, France; 2 School of Anthropology and Museum Ethnography, University of Oxford, Oxford, UK; 3 Harris Manchester College, University of Oxford, Oxford, UK

**Keywords:** evolutionary public health, behavioural ecology, cultural evolution, contraceptive dynamics, fertility, family planning, contraceptive side-effects, demographic transition

## Abstract

Numerous evolutionary mechanisms have been proposed for the origins, spread and maintenance of low fertility. Such scholarship has focused on explaining the adoption of fertility-reducing behaviour, especially the use of contraceptive methods. However, this work has yet to engage fully with the dynamics of contraceptive behaviour at the individual level. Here we highlight the importance of considering not just adoption but also discontinuation for understanding contraceptive dynamics and their impact on fertility. We start by introducing contemporary evolutionary approaches to understanding fertility regulation behaviours, discussing the potential for integrating behavioural ecology and cultural evolution frameworks. Second, we draw on family planning studies to highlight the importance of contraceptive discontinuation owing to side-effects for understanding fertility rates and suggest evolutionary hypotheses for explaining patterns of variation in discontinuation rates. Third, we sketch a framework for considering how individual flexibility in contraceptive behaviour might impact the evolution of contraceptive strategies and the demographic transition. We argue that integrating public health and evolutionary approaches to reproductive behaviour might advance both fields by providing (a) a predictive framework for comparing the effectiveness of various public health strategies and (b) a more realistic picture of behaviour by considering contraceptive dynamics at the individual level more explicitly when modelling the cultural evolution of low fertility.

## Graphical abstract

**Figure:**
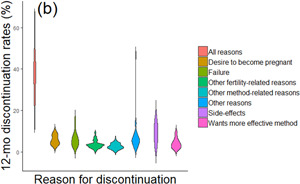

**Social media summary:** Worldwide, millions of women stop using contraceptives. Why? Novel hypotheses from evolutionary public health

## 1. Introduction

Uncovering the processes underpinning the dynamics of contraceptive behaviour is relevant for understanding the demographic transition, defined as the shift from high to low fertility with increasing levels of socioeconomic development in recent and contemporary history (Bongaarts & Watkins, 1996; Borgerhoff Mulder, 1998). Of course, the availability of modern contraception is neither necessary nor sufficient for fertility decline: the European demographic transition started around two centuries before the contraceptive pill was commercialised (Livi-Bacci, 1986) and despite modern contraception being accessible in many parts of sub-Saharan Africa, fertility remains high on average. Yet, as contraceptive technology has been introduced recently in some areas, it is possible to study the cultural evolution of contraceptive use, i.e. the change in the prevalence of contraceptive use at the population level over time. Note that by *cultural evolution* we mean the process of change over time rather than any specific theoretical framework aiming at explaining the evolutionary process (i.e. behavioural ecology or cultural evolution approaches). Given that contraceptive use is linked to reduced infant and maternal mortality, as well as improved socioeconomic opportunities (Ahmed et al., 2012; Canning & Schultz, 2012; Cleland et al., 2012; United Nations, Department of Economic and Social Affairs, 2015), uncovering the mechanisms linking individual behaviour to population change in contraceptive use has important public health implications.

Thus far, evolutionary research on contraceptive behaviour has focused on why people adopt low-fertility ideals and begin using fertility regulation methods, with relatively little attention given to processes underpinning the maintenance of contraceptive behaviour past its initial adoption. Yet, reducing fertility is more than an idea to be accepted once or a process to be initiated. It is an ongoing practice with much variation in execution over time. For instance, contraceptive use can involve a mix of so-called ‘traditional’ and ‘modern’ contraceptive techniques including abstinence, withdrawal, calendar rhythm, condoms, injectables, IUDs, pills, emergency pills and herbal remedies (Marston et al., 2016). In turn, methods differ in their length of action (e.g. short-term vs. long-term), efficacy (e.g. withdrawal is less efficacious than IUD) and perceived cost for future fertility (e.g. non-hormonal vs. hormonal methods leading to side-effects). While family planning studies initially researched the determinants of contraceptive ever-use across methods, there is now a noticeable shift from a focus on ‘*adopters*’ to a concern over ‘*discontinuers*’, i.e. women who stop using a given method of contraception after initiating use (Jain, 2014; Castle & Askew, 2015). There are multiple reasons for discontinuation, and sometimes multiple reasons per discontinuation event, but discontinuation due to side-effects is the most important cause of discontinuation while in need (i.e. stopping despite the desire to continue limiting or spacing their fertility; Ali et al., 2012; Castle & Askew, 2015). The possibility for individuals to both learn from their experience of contraceptive techniques and socially transmit knowledge gained from their positive or negative experiences might have important implications for both contraceptive and fertility dynamics. Further, women's decision to discontinue using contraception (because of side-effects or other reasons) is likely to be shaped by socio-ecological and individual factors (e.g. age, parity, marital and socio-economic status) that influence variation in life-history trade-offs between current fertility and other components of fitness. Thus far, however, the evolutionary human sciences have not yet engaged with the issue of contraceptive discontinuation at the individual level.

This paper discusses the strengths, limitations and future of studies researching the cultural evolution of contraception. While our discussion focuses primarily on modern methods, a similar reasoning could also be applied to traditional methods, even though traditional methods are less efficacious than modern ones for avoiding unplanned pregnancies. We start by briefly outlining evolutionary perspectives on fertility and contraceptive dynamics in high fertility or transitioning populations with widespread availability of family planning services, including behavioural ecology and cultural evolutionary approaches. Second, we suggest ways to harness evolutionary frameworks for understanding variation in the propensity to discontinue contraception due to side-effects (Table 1), the main reason for discontinuation whilst in need. Third, we illustrate how an integration of both adoption and discontinuation dynamics may provide a fuller understanding of the evolution of fertility-regulating behaviour, the transition from high to low fertility and the impact of family programmes on the demographic transition. We conclude by pointing to the importance of individual experience and the flexibility of practices for modelling cultural evolutionary processes.

**Table 1.** Evolutionary approaches to understanding patterns of discontinuation of modern contraception due to side-effects. We focus on discontinuation whilst in need, for which the most common reason is side-effects

Patterns	Ultimate causes	Proximate causes	Hypotheses	Current evidence	Future questions
Diversity in the experience of biological side-effects, i.e. bleeding, headaches, migraines, facial marks and other bodily changes	Evolved dispositions optimising the trade-off between current reproductive effort and future reproduction and survival (immunity).	Reproductive depletion, energetic stress and immune challenges lead to low levels of reproductive hormones. The severity of side-effects may be a function of how high the dose of hormonal contraception is compared with endogenous reproductive hormones levels.	Women will experience more side-effects if they have recently given birth; if they face immune challenges; if they have low levels of energetic resources (living in poverty; experiencing high workload)	Women who gave birth in the last two years are more likely to DSE (Stevens et al., 2020); Women living in poor conditions show lower progesterone levels than better off counter parts (Vitzthum, 2009); Anemic women are more likely to DSE (Stevens et al., 2020); The poorest women report more side-effects (Alvergne et al., 2017; Bradley et al., 2009; Mernissi, 1975; Meskele & Mekonnen, 2014)	Is there a causal relationship between a woman's somatic state before using hormonal contraception and the severity of side-effects?
Diversity in the experience of social side-effects, i.e. stigma or social rejection owing to contraceptive use and associated side-effects	Conflict between the evolutionary interests of women and men and their kin	The discrepancy between the fertility ideals of women and men (and their kin).	DSE is more likely in stratified (high conflict) vs. egalitarian groups (low conflict); in the presence of pronatalist in-laws; if paternal investment is critical to child survival and contraception affects the ability to mobilise paternal investment (through e.g. domestic chores, sexual intercourse)	Women are more likely to choose hormonal methods that afford secrecy in Ethiopia and Ghana (Alvergne et al., 2017; Marston et al., 2016); Women in Cambodia told to D by their husbands if they observed any side-effects (Samandari & O'Connell, 2011); Religious, household and sexual spheres of life were all impacted by hormonal contraceptive use in Bangladesh (Jain et al., 2017)	Is DSE risk predicted by the level of sexual conflict over fertility between and within groups? Does DSE risk covary with perceived infertility stigma? Does DSE risk covary with impact of SE on activities associated with increased paternal investment?
Diversity in the tolerance of side-effects, i.e. variation in the risk of DSE assuming similar severity of side-effects	Evolved dispositions optimising the trade-off between current reproductive effort and future reproduction and survival	SE are perceived to be costly for future survival and reproduction, the magnitude of the cost varying with socioecological factors (disease threat, resource distribution) and individual embodied capital (skill, knowledge, immune function)	DSE is more likely for women suffering the highest cost of SE for future survival and reproduction, such as women at the beginning of their reproductive career; DSE is more likely for women with low embodied capital if SE are perceived to threaten immediate survival	SE are linked to worry about chances of survival in Ethiopia and Uganda (Alvergne et al., 2017; Kibira et al., 2015); SE are linked to worry about reduced future fecundity in many settings (Path, 2015)	Is DSE risk (as opposed to DOR risk) predicted by parity, age and embodied capital? Do SE influence future reproductive (fecundity) and health outcomes (mortality)?
	Evolved dispositions optimising the trade-off between the quantity and the ‘quality’ of offspring	SE are perceived to be costy for existing offspring's embodied capital, the magnitude of the cost varying with the socioecology (disease threat, resource distribution) and individual embodied capital (skill, knowledge, immune function)	DSE is more likely for women with alloparental care and social support; DSE is more likely in polygynous households (competition for paternal investment); DSE is less likely if C is used to avoid costly reproductive overlap with other women in household	None that we are aware of	Does (and which form of) alloparental care and social support increase DSE risk? Does reproductive competition between co-wives increases DSE risk? Do SE decrease levels of paternal investment in offspring?
	Evolved dispositions adjusting the pace of reproduction to the rate of extrinsic mortality	C use is perceived to be costly for reproductive health and future fertility	DSE is more likely in environments with high rates of extrinsic mortality as: (a) high fertility is favoured and (b) benefits to delaying fertility are reduced; DSE is more likely in environments where it ‘pays’ to invest in one's own capital	None that we are aware of	Does DSE risk covary with the rate of extrinsic mortality within and between populations? Does DSE vary across ecologies (e.g. along a rural-urban gradient)?
Diversity in the fear of side-effects, i.e. variation in the transmission of fertility ideals and fear of side-effects through different types of evolved cognitive dispositions for social learning	Evolved cognitive dispositions for payoff-biased social learning	Payoff-biased social learning leads fertility related knowledge to be more attractive; Fertility is a ‘cultural attractor’	Fears of side-effects associated with reproductive function (e.g. infertility) spread faster than other types of fears; Fears are particularly salient for women with the highest cost of infertility	The biggest fear is that a particular method causes infertility (Burke & Ambasa-Shisanya, 2011; Hyttel et al., 2012; Ochako et al., 2015); Fears about infertility from contraceptive use are particularly present among young women with no children (Adongo et al., 2014; Castle, 2003; Hindin et al., 2014; Ochako et al., 2015)	How do fears about SE spread through cultural transmission networks? Are fertility-related fears more likely to lead to DSE? Are women with the highest cost of infertility more attracted to rumours of infertility?
	Evolved cognitive dispositions for kin-biased social learning	Kin-biased social learning leads to the spread of fertility-related knowledge and the maintenance of fertility ideals	Fears spread through kin networks	Negative views of SE by mothers in law influence contraceptive behaviour (Diamond-Smith et al., 2012)	Does fear of SE spread through paternal and maternal kin networks?
	Evolved cognitive dispositions for model-biased social learning	Prestige-biased transmission leads to the spread of fears of SE	Prestige-biased transmission decreases DSE if reducing fertility brings about cultural wealth Prestige-biased transmission favours the spread of DSE if high status individuals are more likely to DSE	Educated urban women in Ghana stopped or avoided hormonal methods to preserve fertility (Marston et al., 2016)	Are women who discontinue perceived as more or less prestigious? Are women more likely to DSE if prestigious network partners DSE?
	Evolved cognitive dispositions for frequency-biased social learning	Conformist-biased transmission leads to the maintenance of fears of SE	Fears are maintained through conformist-biased transmission	Myths are created and reinforced by women's social interactions (Path, 2015) and belief in myths are negatively associated with contraceptive use (Gueye et al., 2015)	Is DSE predicted by community level DSE? Is length of use and S behaviour from specific methods predicted by community method preferences?

C, Contraception; CU, contraceptive use; D, Discontinuation; DSE, discontinuation due to side-effects; DOR, discontinuation due to other reasons; SE, side-effects; S, switch between methods. We propose an evolutionary ecological approach, identifying the ultimate (i.e. evolutionary history and function utility) and proximate (i.e. mechanisms underlying behaviour) factors responsible for patterns of variation.

## 2. Current evolutionary perspectives on the use of modern contraception

Why do people start using modern contraception? Is fertility change driven by a change of ideas and/or a change of economies? Much work has been done in demography and family planning, where two models have been proposed to explain the adoption of fertility-regulating technologies: (a) demand theory (Easterlin, 1975), whereby contraceptive adoption is driven by levels of socioeconomic development and thus responds to a previously unmet need (when a woman does not want to get pregnant but is not using contraception); and (b) diffusion theory (Casterline, 2001), whereby a new idea, technology or behaviour spreads in the population independently of the level of socioeconomic development. A review of 63 developing countries found that, at the aggregate level, family planning programmes both decrease unmet need for contraception by removing ‘barriers’ and increase the demand for contraception through educating people about the benefits of small family size (Bongaarts, 2014), thus providing support for both theories. Yet standard quantitative population-level models of demographic change do not typically consider within-population variation in behaviour that affects fecundity and mortality, nor aspects of cultural transmission that can facilitate the spread of fertility-reducing behaviours (Creanza et al., 2017), limiting the scope of their projections. It has thus been suggested that evolutionary approaches should be integrated into demography (Colleran, 2016), as they focus on what has changed in the environment and what evolved mechanisms underpin fertility and contraceptive dynamics. In this section, we briefly present current evolutionary frameworks, highlighting areas of debate for their integration, and review the empirical evidence for each framework for the adoption (emergence and spread) and the maintenance of modern contraception in a population.

### (a) Toward an integrated evolutionary framework

To understand why a low-fertility variant emerges and spreads, evolutionary scholars have used principles from primarily two frameworks: behavioural ecology (Nettle et al., 2013) and cultural evolution (Mesoudi, 2016). Broadly, a behavioural ecology approach is concerned with the fitness consequences of a given behaviour for the individual and assume that mechanisms do not constrain adaptive responses to the ecology (Winterhalder & Smith, 2000). In contrast, a cultural evolution approach focuses on the social transmission of a cultural variant, often independently of its impact on individual fitness (but see El Mouden et al., 2014). Both approaches assume the dynamics of cultural change over ecological times to be constrained by evolved cognitive dispositions at the individual level, but while BE models are concerned with the impact of behaviour on individual fitness, CE models are concerned with the impact of social transmission biases on the fitness of the cultural trait (e.g. the prevalence of contraceptive uptake at the population level).

Behavioural ecology theory advances that individuals adjust their fertility as a response to an increased perceived trade-off between fertility and investment in their own and their offspring's embodied capital (Kaplan, 1996; Borgerhoff Mulder, 1998; Kaplan et al., 2002). As the risk of death in early childhood decreases, the importance of parental investment for an individual's ability to outcompete their peers increases, especially in skill-based competitive labour markets (Kaplan, 1996), leading to increased trade-offs between fertility and investment in their own and their offspring's capital and ultimately, fertility decline (Mace, 2008). While empirical studies in low-fertility populations have not found lowered fertility rates to be optimal (Kaplan et al., 1995; Goodman et al., 2012), there is some empirical support for a link between variation in fertility trade-offs and inter-individual diversity in fertility (Shenk, 2009; Shenk et al., 2013) and age at marriage in India (Shenk, 2009) or the timing of adoption of modern contraception in Ethiopia (Alvergne et al., 2011; see full review in Colleran, 2016), suggesting that changing fertility trade-offs can promote fertility change. Yet it is also well known to demographers that socioeconomic circumstances alone cannot fully explain the dynamics of fertility behaviour (Cleland, 2001), although the issue remains debated and economic concerns may be of high importance in certain contexts (Colleran & Snopkowski, 2018).

Cultural evolution theorists have proposed that the worldwide phenomenon of fertility decline can be explained by evolved cognitive dispositions for social learning (Richerson & Boyd, 2006). The leading theory posits that if people who delay reproduction become more educated and/or wealthier, and if people imitate the most successful and prestigious, then a fertility-reducing variant could spread (Richerson & Boyd, 1984; Ihara & Feldman, 2004). While direct causal evidence for the role of prestige-biased transmission in spreading fertility reducing behaviour is not yet available, studies at a later stage of the demographic transition found stronger effects for community-level and social-network variables than individual factors in predicting fertility (Colleran et al., 2014) or contraceptive use (Colleran & Mace, 2015). Thus, a comprehensive evolutionary account of fertility decline should incorporate insights from a cultural evolution (CE) approach to traditional behavioural ecology (BE) models (Colleran, 2016).

There is some debate as to whether BE and CE models are compatible, limiting the scope for their integration. Cultural models can be seen both as mechanistic models of how fertility declines or as causal models for why fertility declines (Mattison & Shenk, 2020), but there is significant debate as to whether CE offers a proximate (Scott-Phillips et al., 2011) or an ultimate (Laland et al., 2011, 2014; Bateson & Laland, 2013) explanation. To evaluate the conditions under which CE and BE models are compatible it is helpful to (a) distinguish between the impact of behaviour on individual fitness from its impact on the dynamics of a cultural trait, (b) separate out the phases of the dynamics of the cultural evolutionary process (emergence, spread and maintenance) (Alvergne et al., 2011; Colleran et al., 2014) and (c) interact rather than add socioeconomic and cultural factors. First, while CE models are not concerned with the impact of behaviour on individual fitness, both BE and CE models can be used to explore the forces underpinning the dynamics of behaviour change over ecological scales (Mattison et al., 2018). Second, while social learning dispositions might not be directly relevant to explaining why a ‘low-fertility variant’ emerges in the population in the first place, as there is no existing model to imitate, CE models are not incompatible with BE models showing that, in environments where wealth is heritable and a better predictor of long-term fitness than fertility, material motivations could be selected over pure reproductive motivations (Rogers, 1995). Indeed, CE models view the emergence of a negative correlation between fertility and wealth as the starting point for the spread of low fertility. Third, with regards to the spread and maintenance of behaviour, BE and CE models could be offering alternative, independent or complementary explanations depending on whether the process of cultural transmission interacts with individual fertility trade-offs. While both approaches are often considered alongside each other (Borgerhoff Mulder, 2009; Shenk, 2009; Alvergne et al., 2011; Shenk et al., 2013; Colleran et al., 2014; Snopkowski & Kaplan, 2014; Colleran & Mace, 2015; Howard & Gibson, 2017; Mattison et al., 2018), integration remains limited.

### (b) The adoption and spread of modern contraception

Most evolutionary accounts of contraceptive dynamics have focused on identifying the forces underpinning the adoption, or first use, of modern contraception. Studies have been conducted in various settings, from high-fertility to transitioning to low-fertility populations, and predictions from both BE and CE are helpful in advancing knowledge of contraceptive dynamics. From a BE perspective, it is not clear that the initial adoption of modern contraception is driven by the life-history trade-off between fertility and child survival (i.e. so-called quantity-quality trade-off, reviewed by Lawson & Borgerhoff Mulder, 2016): women working in the cash economy predominate among ever-users among the Pimbwe, Tanzania (Borgerhoff Mulder, 2009) and contraceptive use does not improve child survival among the Arsi Oromo, Ethiopia (Alvergne et al., 2013). Some data rather suggest that contraceptive adoption and use are a response to strong life-history trade-offs between current and future reproduction, which can be increased owing to, e.g., maternal somatic depletion or changes in age at first birth, maternal education or maternal marital status. Modern contraception is often used to space births in high-fertility and African contexts (Mace et al., 2006; Borgerhoff Mulder, 2009; Alvergne et al., 2013), and in rural Gambia, the technology is used to recuperate between births and improve body resilience for future reproduction (Bledsoe et al., 1998). Further, early adopters are more likely to be fertile women (Mace et al., 2006; Alvergne et al., 2013) with higher parity for their age (Mace & Colleran, 2009; Alvergne et al., 2011, but see Borgerhoff Mulder, 2009), possibly suggesting that early adopters are more physiologically ‘depleted’ than others and/or on the highly fertile end of the spectrum.

From a CE standpoint, the slow spread of modern contraception in the early stages of the demographic transition points towards the absence of prestige-biased social transmission (Alvergne et al., 2011), suggesting that contraceptive uptake does not take off if the accumulation of cultural wealth (e.g. education) is independent from fertility (e.g. reducing fertility does not allow the gaining of wealth). Yet it is difficult to conclude the absence of social transmission when only semi-complete networks are available. There is also little evidence that kin influence goes against contraceptive uptake (the ‘kin influence hypothesis’; Newson et al., 2005): contraceptive use is either more likely among women with matrikin (in Tanzania; Borgerhoff Mulder, 2009), with extended kinship ties (in Thailand; Godley, 2001) and whose female kin are users (in Poland; Colleran & Mace, 2015), or is independent from the presence of kin (in Gambia; Mace & Colleran, 2009). Non-parental transmission of contraceptive choices is found to be important further along the demographic transition, in rural Poland (Colleran & Mace, 2015) and Bangladesh (Gayen & Raeside, 2010) for instance, and with modernisation (Kohler et al., 2001). Thus, evolutionary frameworks offer a strong theoretical backdrop against which to propose causal factors of change in the prevalence of contraceptive ever-users in each population.

### (c) The discontinuation of modern contraception

There has been little attempt to harness evolutionary perspectives for understanding patterns of diversity and changes in the prevalence of contraceptive discontinuation within and among populations (Vitzthum & Ringheim, 2005). However, the discontinuation of contraceptive use is increasingly becoming one of the most significant concerns for family planning programmes: one in five ‘ever-users’ discontinue modern methods of contraception while still in need of family planning worldwide (Jain et al., 2013). At the individual level, discontinuation while in need puts women at risk of unwanted pregnancies and unsafe abortion (Sedgh et al., 2014). At the population level, contraceptive discontinuation produces a ‘leaking bucket phenomenon’ (Jain, 2014), whereby programmes aiming at increasing the prevalence of contraceptive use are hindered by contraceptive discontinuation. The reproductive consequences of contraceptive discontinuation while in need are substantial: more than 30 million unintended pregnancies occur among women not using contraception (Ali et al., 2012). Thus, following Jain's claim (Jain, 2014) that family planning programmes would do better to concentrate on retaining existing users rather than focus on recruiting new clients, recent research and policy reports have suggested that tackling contraceptive discontinuation is central to achieving public health goals (Ali et al., 2012; Jain et al., 2013; Castle & Askew, 2015).

Possible reasons for the lack of discussion of contraceptive discontinuation in the evolutionary human sciences are: (a) studies of contraceptive use test hypotheses relevant to explaining the Darwinian puzzle of fertility decline rather than take contraceptive technologies as an object of study in its own right, and indeed theoretical work has researched the importance of biased social transmission for explaining the maintenance of low fertility in industrial populations (Ihara & Feldman, 2004; Kendal and Ihara, 2005; Ghirlanda & Enquist, 2007; Kolk et al., 2014); and (b) cultural evolution models are adapted from population genetics models (Cavalli-Sforza & Feldman, 1981), in which a trait, once inherited or acquired through social transmission, is not typically lost at the individual level. Such models appear well adapted to long-acting, less easily reversible contraceptive methods (sterilisation, IUDs, implants) used for fertility limitation, but do not apply well to the use of short-acting methods, such as the pill or injectable, used for fertility timing/spacing; (c) Cultural evolutionary studies of fertility change tend to focus primarily on the social transmission of information rather than information obtained from one's experience (e.g. of contraceptive use). From a theoretical perspective, however, both CE and BE models can provide predictions for why and under which conditions women might discontinue contraception (Table 1). While some individuals might discontinue contraception in order to reproduce in line with their reproductive ideals, for others, contraceptive discontinuation while in need (e.g. because of side-effects) is likely to be at odds with a woman's fertility intentions. Below we outline how public health data on contraceptive discontinuation whilst in need can be analysed within an evolutionary framework.

## 3. Towards an understanding of the evolutionary dynamics of contraceptive discontinuation whilst in need

Why do individuals discontinue contraception despite wanting to delay or prevent pregnancy (i.e. whilst in need)? Women report multiple reasons for discontinuation whilst in need including method-related dissatisfaction due to concerns about side-effects (hormonal methods) and efficacy (condoms, withdrawal), fears or rumours that hormonal contraceptives cause cancer and/or infertility, partner's desired fertility, issues of cost and availability, as well as community-level contraceptive prevalence and attitudes (Campbell et al., 2006; Bradley et al., 2009; Ali et al., 2012; Castle & Askew, 2015). Whilst issues of cost, availability and partner's desires do influence women's decision to discontinue, they are much less frequently given as reasons for discontinuation for almost all method types than side-effects and health concerns (Bellizzi et al., 2020), suggesting that the latter reasons are the most salient drivers of discontinuation for the majority of women. In this line, data from low- and middle-income countries show that contraceptive side-effects and health concerns are the leading cause of discontinuation whilst in need (Ali & Cleland, 1995; Blanc et al., 1999; Castle & Askew, 2015). Reported physiological side-effects from hormonal contraception include heavy bleeding, irregular bleeding, nausea, depression and loss of libido, amongst others. Injectable contraceptives are consistently cited as the method with highest rates of discontinuation due to side-effects and health concerns; however, qualitative studies show that women also report significant burdens of side-effects from pills, implants and IUDs (Meskele & Mekonnen, 2014; Chebet et al., 2015; Jain et al., 2017). A large body of qualitative literature show that women find side-effects highly problematic (Samandari & O'Connell, 2011; Meskele & Mekonnen, 2014; Ochako et al., 2015; Capurchande et al., 2016; Jain et al., 2017), as exemplified by the quote from a Ugandan woman: ‘I was bleeding so much, I thought I was going to die’ (Kibira et al., 2015).

From an evolutionary perspective, discontinuation due to the experience and/or fear of side-effects can be reframed as the outcome of ultimate causes and associated proximate mechanisms, in particular (a) evolved physiological mechanisms adjusting reproductive function to one's socioecological circumstances and causing variation in susceptibility to side-effects, (b) evolved cognitive dispositions adjusting reproductive behaviour, including discontinuation owing to side-effects, to context-specific fitness costs and benefits and (c) evolved cognitive dispositions underpinning social learning. Further, these forces might act independently or interact, e.g. the experience of side-effects, such as heavy bleeding or lack of bleeding, may fuel rumours about infertility. Below we outline how an evolutionary approach can shed new light on the dynamics of discontinuation due to side-effects (Table 1).

### (a) Patterns of diversity in the experience of biological side-effects

With regards to contraceptive discontinuation, the question of why some women experience more side-effects than others has, to date, attracted little attention from either clinical trials (Inoue et al., 2015), which focus on overall burden rather than interindividual variation, or public health, which concentrates efforts on dispelling misconceptions (Path, 2015). In contrast, reproductive ecologists have suggested that the risk of experiencing side-effects may be higher for women living in socioecological settings characterised by poverty, owing to low endogenous reproductive hormone levels relative to the contraceptive doses received (Bentley, 1994; Vitzthum & Ringheim, 2005). Indeed, previous anthropological studies have shown that levels of reproductive hormones vary significantly across cycles, between women and across populations (Jasienska & Jasienski, 2008), with lower levels often (but not always) found in less affluent contexts (Vitzthum, 2009). In this line, an analysis of Demographic and Health Survey (DHS) data from Ethiopia shows that the risk of contraceptive abandonment is 42% higher among women living in poverty – an effect also reported in qualitative interviews e.g. ‘Only women having access to better diet [egg, meat, butter …] should take pills or injectables as it does not work for those having poor diet’ (Alvergne et al., 2017; see also Meskele and Mekonnen, 2014). The perceived relationships between wealth and discontinuation owing to side-effects has also been shown in other settings such as Morocco, where women state they will use the pill when they can ‘afford to have a banana and glass milk from time to time’ (Mernissi, 1975). In the same lines, using DHS data from Ethiopia, we found that anaemia increases the risk of discontinuing using contraception owing to side-effects (DSE), but not the risk to discontinue owing to other reasons (DOR), suggesting that poor somatic condition predicts the experience of negative side-effects (Stevens et al., 2020). Yet the hypothesis that current doses of hormonal contraceptives may be maladjusted to some women's bodies has not been directly tested to date (Jasienska et al., 2017).

### (b) Patterns of diversity in the experience of social side-effects

The experience of physiological side-effects can in turn elicit social side-effects, i.e. additional consequences that have a negative impact on social relations. The nature and severity of these effects depend on the local cultural context. For instance, variation in libido and mood changes have various implications depending on the cultural expectations of a woman's intimate relationships. In Kenya, women report that injectables make users ‘cold’, leading to a lack of sexual arousal and raising suspicions of infidelity from husbands (Ochako et al., 2015). Another side-effect that can have serious social consequences is disrupted menstruation, often subject to many local taboos and norms. In Mali, menstrual disruption can lead to accusations of witchcraft or immoral behaviour and can prevent a woman from preparing food and praying (Castle, 2011). In Bengali society in Bangladesh, after menstruation, a woman must wait 7 days before she can resume her prayers. She must avoid sexual intercourse when menstruating, as there is a belief that contact with menstrual blood can jeopardise a man's sexual and spiritual integrity (Rashid, 2001). Such social side-effects can lead to reduced indirect paternal investment (e.g. investment in a child through support to the mother; Geary, 2015), as well as loss of social support, which in turn decreases the potential fitness benefits of delaying reproduction. If side-effects are perceived to entail costs for one's future ability to conceive and/or in terms of loss of resources owing to social exclusion or decreased paternal investment, then the short-term costs of continuing contraceptive use might be perceived to outweigh the long-term benefits of using hormonal contraceptives to delay or space fertility. This is particularly relevant in some high-fertility contexts where hormonal contraception is primarily used to space births and conserve strength (Bledsoe et al., 1998; Alvergne et al., 2013). Thus, physiological side-effects must be considered given the socio-cultural contexts of contraceptive users.

To understand diversity in the severity of social rejection owing to contraceptive use and associated side-effects, sexual conflict theory (Borgerhoff Mulder & Rauch, 2009) might prove helpful. When the reproductive interests of men and women do not align, with men favouring higher fertility than women, conflict over contraceptive use and side-effects might ensue. One would predict that social side-effects might be more important in social systems associated with sexual conflict over fertility (stratified or unequal groups as compared to egalitarian groups; Borgerhoff Mulder & Rauch, 2009). Further, sexual conflict over reproductive interests extends from husbands and wives to their kin, and thus one would expect the risk of discontinuation due to side-effects (DSE) to be modulated by the presence of kin. A promising avenue could be to compare DSE risk across and within social groups varying in their kinship ecologies and in degree of sexual conflict over fertility.

### (c) From side-effects to contraceptive discontinuation: patterns of diversity in the tolerance of side-effects

From a behavioural ecology perspective, much of contraceptive discontinuation whilst in need can be seen as the outcome of the impact of side-effects on perceived fertility trade-offs. First, individuals face a life-history trade-off between current reproductive effort and future survival and reproduction. Thus, DSE can be understood as a form of investment in future survival and reproduction at the expense of current effort in offspring and own capital. Within this framework, DSE is expected to be more likely for women suffering the highest cost of side-effects for future survival and reproduction, for instance women at the beginning of their reproductive career: women who have never had a baby will be more likely to discontinue while older women will be more likely to tolerate side-effects. Conversely, if side-effects are perceived to threaten one's immediate chances of survival, which is more likely to be the case for women suffering the strongest side-effects, then the cost of continuing contraceptive use will outweigh the benefits of delaying or spacing fertility. In a study we conducted in Ethiopia, the experience and fear of physiological side-effects of the injectable contraceptive in the form of irregular and heavy bleeding led women to worry about significant threats to their health (the words used for heavy bleeding were synonymous with those used for ‘loss of life’) and their fertility (resulting from a delay to return to fertility after use or amenorrhea and/or a change in bleeding patterns while using). Thus, women experience various trade-offs depending on their circumstances and life-histories, and those need to be considered for predicting the risk to DSE.

Second, individuals face a life-history trade-off between fertility and survival of existing offspring (Lawson & Borgerhoff Mulder, 2016). If DSE is a form of investment in fertility at the expense of offspring quality, it might be more likely if alloparental care is available. Alloparental care is key to increasing family size (Sear & Coall, 2011) and correlates with earlier reproduction among the Mosuo (Mattison et al., 2014), suggesting that alloparental care reduces the cost of reproduction. DSE is also potentially influenced by female reproductive competition. If side-effects interfere with marital relationships, and thus jeopardise women's ability to compete for paternal investment, then DSE might be more likely among polygynous households. Alternatively, if women use contraception to avoid the cost of a reproductive overlap with other women in the household (i.e. mother-in-law and co-wives, mother and sisters, depending on the social system), then tolerance to side-effects might be increased.

Third, the strength of such trade-offs is likely to vary across populations, between individuals and over the lifespan, depending on context (Uggla, 2020). For instance, individuals are expected to adjust their reproductive scheduling to the local rate of extrinsic mortality (i.e. a risk independent from individual phenotype and applying equally to all members of a population (Charnov, 1993), which vary across countries or neighbourhoods; reviewed in Uggla, 2020). When the risk of extrinsic mortality is high, the fitness benefits to delaying fertility are reduced and thus all else being equal, DSE might be more likely as compared with environments where child survival is highly dependent on parental investment. In addition, if the fitness benefits associated with investing in one own's capital depend on the subsistence economy (i.e. subsistence economy vs. competitive labour market economy), tolerance for side-effects in order to delay age at first reproduction is likely to be increased when education positively correlates with wealth. Macro-level features of the socioecology are thus also relevant to explaining patterns of DSE.

### (d) Diversity in the fear of side-effects

Public health does not often make the distinction between women who experience side-effects and those who fear them. Rather, fear of side-effects is often referred to as misinformation, misconceptions or rumours, with an underlying assumption that many fears are not founded in real experience (Chipeta et al., 2010; Hindin et al., 2014; Path, 2015). In turn, dispelling myths (e.g. contraceptives lead to health problems; Gueye et al., 2015) through education remains the preferred strategy for tackling discontinuation. However, one must acknowledge that experience and rumour are not mutually exclusive and can often interact (Diamond-Smith et al., 2012). Women's discussions of their own true experience of contraceptive side-effects end up creating fears based in the real experience of previous users. These rumours can then have significant impact on the rates of discontinuation. In this line, one study from three low- and middle-income countries (Nigeria, Kenya, Senegal) found a positive association between the aggregate level of method use and the prevalence of negative myths in a community in Nigeria (odd ratio, 1.6; Gueye et al., 2015) suggesting that myths originate from experiences. From an evolutionary perspective, this is not suprising, as humans (and other animals) rely on a complex mix of individual and social learning mechanisms to make decisions (Mesoudi, 2016). Understanding which mechanisms are at play in shaping cultural transmission in a given ecology is critical for predicting the dynamics of discontinuation over space and time.

Fear of side-effects when using hormonal contraception do appear to be prevalent for all methods and all groups of society (Path, 2015), suggesting that the motivation underpinning contraceptive discontinuation is culturally transmitted. In this line, the transmission of side-effect experiences and their impact on contraceptive behaviour has been documented in qualitative studies conducted in Kenya (Rutenberg & Watkins, 1997; Kohler et al., 2001; Ochako et al., 2015), Egypt (DeClerque et al., 1986), the Dominican Republic (Porter, 1984) and Ethiopia (Alvergne et al., 2017). Women learn about side-effects from their social networks, and peers and other community members act as their main sources of information over and above health providers and other educational sources (Porter, 1984; Rutenberg & Watkins, 1997; Castle & Askew, 2015; Ochako et al., 2015). Of course, the social transmission of contraceptive behaviour may be limited in some contexts owing to the high level of stigma associated with the practice (Alvergne et al., 2017). Yet, how fears about contraceptive side-effects and subsequent discontinuation behaviour may transmit through a network will vary based on social and ecological contexts. An evolutionary framework can be useful for making predictions about the dynamics of contraceptive discontinuation behaviour and transmission mechanisms in different contexts. To advance this research agenda and disentangle which processes are at play, we have suggested below questions needing investigation.

First, fears about infertility may persist as a cultural trait owing to pre-existing cognitive biases (Sperber, 1996). For instance, fears about contraception as a threat to future fertility is one of the most persistent and ubiquitous fears about modern contraception worldwide. This continuity may not be simply coincidental and may possibly be based on an existing cognitive bias to avoid risks to future fertility. Sperber (1996) has argued that the stability of a cultural trait may emerge as people independently reconstruct representations based on pre-existing cognitive biases, or ‘attractors’ (Sperber, 1996; Morin, 2015). This concept has been used across the disciplines of anthropology, psychology and linguistics to explain the surprising consistency of cultural traits (Buskell, 2017). Fears of contraception-caused infertility may be a cultural attractor, which may explain its ease of social transmission and surprising persistence across time and geography, despite education efforts within family planning programmes. This suggests that in order to counteract the spread of rumours, efforts should be directed at identifying which specific side-effects are associated with a fear of infertility and reducing them.

Second, the mechanisms underpinning the social transmission of contraceptive behaviours might vary as a function of local desired fertility norms. The saliency of fear of possible infertility will be stronger where the local cultural importance assigned to fertility is high, and where social influence mechanisms (e.g. frequency-biased social learning) might favour the spread and maintenance of discontinuation. This may be the case among women living in sub-Saharan Africa where the ideal family size is high, and where fertility decline appears to have stalled (Bongaarts & Casterline, 2013). Further, if non-users or discontinuers are perceived to achieve higher wealth (unpublished interviews in Ethiopia reveal that irregular heavy bleeding owing to contraceptive use prevents some women from attending university or employment) and fertility, they may become seen as prestigious individuals and the spread of discontinuation through prestige-bias transmission might ensue.

Third, cultural transmission is also driven by social learning, where an individual's circumstances will interact with the process of diffusion. For instance, payoff-biased social learning, where an individual copies the behaviour with the highest observable payoff, leads to the quickest spread of the most successful techniques in a wild primate species (Barrett et al., 2017). Learning about contraceptive side-effects is not independent from fitness related information as qualitative studies have shown that fears about infertility from contraceptive use are particularly present among young women with no children in contexts which place high value on fertility (Castle, 2003; Adongo et al., 2014; Hindin et al., 2014; Ochako et al., 2015). Social network studies interacting individual characteristics with cultural transmission networks might be able to evaluate variation in how individuals make use of various learning strategies.

Applying evolutionary models to the study of contraceptive discontinuation can help provide explanations for patterns of discontinuation across cultural, social and other ecological contexts. Such models can be used to predict *whether* and *when* women discontinue hormonal contraception. Indeed, the timing of fertility – age at first birth, age at last birth and inter-birth intervals – is an important component to fitness as fertility poorly predicts fitness in a non-stationary population (Jones & Bird, 2014). To date, however, scholarship on the cultural evolution of fertility decline has not sufficiently considered (1) that behavioural change is an incremental and dynamic process (e.g. adopt, stop, re-adopt), rather than the outcome of a binary choice, and (2) that the experience of contraceptive use, be it positive or negative, can be socially transmitted, bringing contraceptive adoption, discontinuation and switching in full circle. In Table 1, we draw on and integrate evolutionary approaches to propose future research questions and data to be collected for understanding patterns of discontinuation whilst in need, particularly due to side-effects. Note that before this can be done, however, a tool for measuring the severity of side-effects in a non-clinical setting is required for each context.

## 4. From contraceptive to fertility dynamics

**Figure 1.**
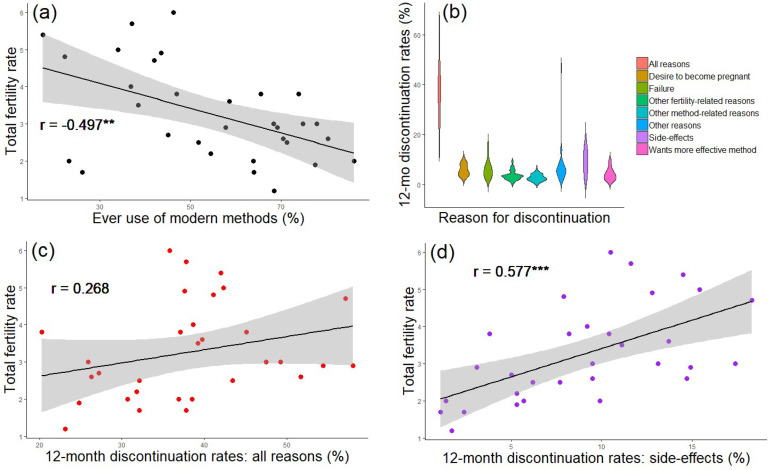
Contraceptive dynamics and fertility. (a) Scatterplot of total fertility rate and ever use of modern methods of contraception. (b) Violin plot of 12 month discontinuation rates by reason for all women using any modern method. Discontinuation due to side-effects is one of the top reasons for discontinuation. (c) Scatterplot of total fertility rate and 12 month discontinuation rate due to all reasons. (d) Scatterplot of total fertility rate and 12 month discontinuation rate due to side-effects. There is a strong positive correlation between total fertility rate (TFR) and (d) discontinuation due to side-effects, stronger than that between (c) TFR and both discontinuation due to all reasons and (a) contraceptive ever-use. Note that these are crude associations and additional multivariate analysis would help elucidate these effects further. Data is taken from aggregate DHS results, accessed via https://www.statcompiler.com/en/ on 21/04/20. A total of 31 low- and-middle-income countries were included in the analysis, with surveys spanning the years 1990–2015. For all countries included, only the most recent survey which had available data on all variables used was included. All data is for all (married and unmarried, results are the same for married only) women and only refers to modern methods. The R script and data are available in Online Appendix 1. *R* values indicate the results of Pearson's product-moment correlation tests. *** *p* < 0.001. ** *p* < 0.01.

Whilst contraceptive continuation is neither necessary nor sufficient to cause demographic transitions, and other methods of fertility regulation are important to fertility decline, contraceptive behaviour does have the potential to impact the pace of fertility decline. In this section we (a) review existing models of the relationship between the cultural evolution of contraceptive behaviour and demography, (b) sketch a new framework for modelling the evolution of contraceptive behaviour to consider change within an individual's lifetime and (c) discuss the implications of considering contraceptive dynamics and its impact on fertility for family planning policy. We argue that understanding how contraceptive uptake, discontinuation and switching between methods interact to shape fertility dynamics is necessary to provide a better understanding of how contraceptive behaviour influences fertility (Figure 1).

### (a) Existing models on the demographic impact of the cultural evolution of fertility

In the field of cultural evolution, previous studies of the interaction between demography and cultural evolution have aimed at understanding how changes in population size alter the evolution of cumulative culture and the maintenance of cultural complexity (Henrich, 2004; Ghirlanda & Enquist, 2007; Enquist et al., 2008; Ghirlanda et al., 2010). Only a few studies have proposed a mechanistic model to study the impact of cultural evolution on fertility and the demographic transitions (Ghirlanda & Enquist, 2007; Fogarty et al., 2013; Kolk et al., 2014). Those have been concerned with explaining the maintenance of low fertility because of rapid cultural change that increases the amount of non-parental transmission. In those models, however, contraceptive dynamics at the individual level (adoption, switching between methods, discontinuation) are not considered. More generally, while discontinuation or ‘culture loss’ has been previously modelled (e.g. Henrich, 2004), it usually occurs at the population level and results from a cultural transmission error. Here we argue for the need to consider contraceptive dynamics at the individual level, which echoes other calls for a ‘systems approach to cultural evolution’ (Buskell et al., 2019).

In the public health literature, only a few statistical models have formalised the evolving relationship between individual contraceptive behaviour and population level outcomes (contraceptive prevalence and total fertility rate). Some focused on the impact of method mix on switching and contraceptive prevalence (reviewed in Blanc et al., 1999). If increasing method mix enables more switching between methods and leads to less discontinuation, contraceptive prevalence is expected to increase (Castle & Askew, 2015). Others investigated the reproductive consequences of contraceptive discontinuation (Blanc et al., 1999, 2002; Jain et al., 2013). A study of DHS data from 15 countries (Blanc et al., 1999) estimated that the total fertility rate would decrease by 20–48% in the absence of discontinuation while in need of contraceptives. However, those predictions do not consider the possibility either that negative experiences of contraceptive use are socially transmitted or that the risk to discontinue depends on individual circumstances. More generally, the mechanisms linking individual to group levels patterns are poorly understood.

### (b) The evolution of contraceptive strategies

To better understand the mechanisms underpinning observed patterns of contraceptive discontinuation given socioecological contexts, it might be helpful to extend adaptive models of human *reproductive* decision-making to human *contraceptive* decision-making. When should one change contraceptive behaviour? Behavioural ecology and cultural evolution models potentially make different and complementary predictions for observed patterns. Following Mace (1998), one could consider a behavioural ecology state-dependent dynamic model on the assumption that contraceptive behaviour is adaptive. In such a model, parents would decide when to have another baby as a function of the environment they currently live in. Decisions would depend on the state of the parent, which could be described at any time by three variables: the type of contraceptive used (a proxy for the fitness cost of increasing the interbirth interval if the type of contraceptive causes side-effects perceived to have a reproductive health or fertility cost), the level of reproductive hormones (a proxy for the risk of experiencing side-effects) and the number of existing offspring. Optimal decision rules or contraceptive strategies would be those that maximise the number of grandchildren. The study of the discrepancy between observed and predicted patterns of discontinuation would inform on the importance of individual learning for contraceptive behaviour.

From a cultural evolution perspective, a classic population genetics model could be extended following Boyd and Richerson (1985) and Newson et al. (2007) to understand the evolution of cultural norms given the impact of natural selection on the transmission of cultural variants. For instance, one could consider a polymorphic cultural trait, e.g. ‘belief that using modern contraception leads to infertility’ and ‘belief that using modern contraception improves fertility potential’ and investigate the impact of various transmission biases (e.g. cultural selection) during social interactions on the evolution of norms over successive generations. Which learning biases lead to the maximisation of individual fitness? Depending on the transmission mechanism at play, what is the shape of the diffusion curve? The study of the discrepancy between observed and predicted patterns of discontinuation would inform on the importance of social learning for contraceptive behaviour.

### (c) The impact of contraceptive strategies on demography

Once evolutionary processes have been studied and mechanisms better understood, ecological processes at the timescale of a generation should be considered if one is to consider the impact of contraceptive behaviour on population demography. To that end, epidemiological-like compartment models, such as SIS (susceptible-infected-susceptible) models (Anderson & May, 1991), could present an interesting alternative to population genetics-like models (Cavalli-Sforza, L.L. & Feldman, 1981) for the study of the evolution of culture and its ecological impacts (Cavalli-Sforza & Feldman, 1981; Lewens, 2015). Compartments models would allow the consideration of contraceptive behaviour as a process, i.e. that individuals can change state throughout their lives, and if contraceptive behaviour is linked to vital dynamics (e.g. birth and death rates), an estimation of the impact of contraceptive dynamics on demography would be possible. A ‘multiple strains’ SIS model should be favoured to account for the diversity of contraceptive methods and estimate their ‘virulence’, i.e. how well they might spread. However, compartment models, in their simplest forms, are deterministic, and thus do not allow for the consideration of interindividual variability in ‘infection’ risk (i.e. adoption), ‘recovery’ (i.e. discontinuation) and interactions rates. To address those issues, epidemiologists have various tools at their disposal, including (a) the shift from deterministic to stochastic compartmental models, which introduce variability or white noise in parameters and (b) network models, which are also stochastic but offer a flexible framework for repeated contacts and for considering micro-level behaviour such as partner choice based on sex or age. Within network models, the classic ‘metapopulation’ approach, where interactions are more frequent within than between ‘patches’, can be used to divide the population according to sociological parameters (e.g. age, experience of side-effects, income, opinion; Kitchovitch & Liò, 2011; Gargiulo & Huet, 2010). Another possibility is to use agent-based models, which probably offer the most flexible approach. Previous research suggests that stochastic epidemic metapopulation model and agent-based models give similar results, but the relevance of each approach depends on the particular aspect of the evolutionary dynamics to be studied and the availability of real-world data (Chen et al., 2018).

### (d) Policy strategies

Modelling contraceptive dynamics as an evolutionary and ecological process provides a starting point to formally investigate the outcomes of various family planning strategies. This approach could be used to compare the efficacy of different strategies to promote the demographic transition such as encouraging switching (e.g. increasing the number of methods available) and improving contraceptive technology (e.g. to reduce side-effects) compared with investing in recruiting new adopters (e.g. acquiring new clients). The current public health default strategy is to increase modern contraceptive prevalence (mCPR) by focusing on counselling. For instance, the UNFPA cites the lack of knowledge and incorrect perceptions about the health risk of contraceptive methods as the main causes for discontinuation (UNFPA, 2019). In turn, many family planning programmes assume that providing full and consistent information about the safety of contraception will lead to 'rational' decisions (from the point of view of family planning programmes), leading people to ignore their fears of side-effects and take up contraception (Kaler, 2009). However, a strategy that focuses on health education about the safety of contraceptive use might not be effective if it contradicts users’ experiences.

One strategy often cited to reduce unmet need includes increasing switching between methods through augmenting method mix. FP2020 cites commitment to improving access to long-acting reversible methods (LARMs) (FP2020, 2017). Yet this strategy is not independent from the common metric used for evaluating FP programmes, couple years protection, which might favour LARMs (Darroch & Singh, 2011). Some authors have questioned the motives of LARM promotion in trying to achieve policy objectives over thinking about women's choice (Gomez et al., 2014). Recent studies also reveal that health providers sometimes push for LARMS despite women requesting using short-acting reversible methods (Yirgu et al., 2020). Such a strategy might discourage women taking up contraceptives altogether, as it is often difficult to get long-acting methods removed (e.g. IUDs and implants).

An alternative strategy would be to improve current methods. It has been suggested that reducing side-effects may be achieved by changing either the dose of contraceptives or women's physiology. Vitzthum and Ringheim (2005) note that there may be a biological basis (perhaps related to diet, nutrition or other metabolic factors) for variation in women's experience of side-effects and tolerance of hormonal contraception. In this line, women given much lower doses of oral contraceptives in both Latin America and Thailand had lower rates of irregular bleeding and few side effects, leaving them less likely to discontinue (Koetsawang et al., 1995). The recent roll out of Sayana Press, a lower-dose short-term acting injectable, might thus be a promising avenue in tackling discontinuation.

While many countries and family planning programmes focus on increasing mCPR, shifting the focus to tackling unmet need might prove both more efficacious and more ethical. Indeed, women who do not express a desire to limit family size or prefer to space births may be making ‘rational’ choices, from an evolutionary or biocultural perspective, given their local circumstances and the impact of the technology on their immediate health, resources and/or social circumstances. Future public health efforts should aim at providing women who need contraceptives with the right method for their goal (space, stop, delay) and their sociocultural and biological contexts (Cates & Maggwa, 2014). As compared with pushing the family planning agenda to increase new adopters or promote only LARMs in order to achieve mCPR targets, providing the conditions and the acceptable technologies for women to invest in themselves and their offspring might be more effective in achieving the demographic transition or other policy goals such as decreasing infant mortality and improving child growth and maternal health.

## 5. Conclusions

Understanding patterns of contraceptive discontinuation has implications for predicting fertility dynamics and the demographic transition. Evolutionary scholars have yet to consider this issue when modelling the spread of low-fertility ideals, and public health models, aimed at estimating progress towards reducing unmet need for contraception (Kantorová et al., 2020), place the focus heavily on understanding adoption rather than continuation. In this paper, we call for evolutionary demography (Sear et al., 2016; Sear & Burger, 2020) and public health studies to go beyond adoption dynamics when modelling behaviour, because contraceptive discontinuation while in need opposes and possibly changes fertility ideals with demographic consequences. Indeed, fertility ideals are often renegotiated in light of changing circumstances (e.g. schooling of women; Behrman, 2015), and thus fertility ‘strategies’ are perhaps best understood as flexible (Trinitapoli & Yeatman, 2018; Hruschka et al., 2019). We suggested ways in which an evolutionary framework can be harnessed to uncover the mechanisms underpinning patterns of discontinuation across groups, time and space. One of the most pressing types of data needed for this is a measurement tool for the prevalence and severity of side-effects in a non-clinical setting, which previously have only been documented qualitatively. We also outlined possible pathways for modelling both the biological evolution of flexible contraceptive behaviour strategies and the cultural evolution of modern contraception as a technology. While public health models aimed at assessing the demographic impact of alternative family planning strategies might gain from situating contraceptive behaviour within an evolutionary ecological framework, the evolutionary human sciences might also benefit from considering ‘culture loss’ or the reversibility of behaviour at the individual level more explicitly when modelling the cultural evolution of low fertility.

Considering behavioural discontinuation can also more widely inform evolutionary debates over the spread of low fertility. For instance, it has been proposed that institutions associated with monogamous marriage suppress intra-sexual competition and reduce total fertility, which lead them to be favoured by group selection because they promote success in inter-group competition (Henrich et al., 2012). This paper shows that individual learning from bodily experience (also referred to as embodied, somatic or visceral learning; Lewens, 2015), may, given individual socio-ecological circumstances, produce a transmission bias against the cultural evolution of low fertility. If individual learning constantly fuels within-group variability in fertility behaviour, then the conditions for cultural group selection (according to the Price equation, between-group variability should be larger than within-group variability; El Mouden et al., 2014) might not be met. Empirical data on the social transmission of discontinuation are needed to evaluate this possibility further.

Following others who have argued that cultural evolution need not imply group selection (Amir et al., 2016), we find that culture is more than socially transmitted information and can evolve following changes in life-history trade-offs (owing to e.g. the use of a particular technology, a change in individual or socio-ecological circumstances). This is not to say that social transmission is not crucial (see Whiten et al., 2016 for a review of cultural diffusion in humans and other species). The idea that an individual negative experience can counteract cultural pressure has been raised by others in a formal model showing that cultural evolution that reduces genetic fitness can be evolutionarily unstable (El Mouden et al., 2014). Considering behavioural traits as practices that can be discontinued, and understanding the conditions under which discontinuation occurs, might have implications for understanding the evolution of other costly behaviours or Darwinian puzzles. The commonality of discontinuation behaviour remains to be investigated, but we hope that this paper will encourage future studies within the fields of cultural evolution and behavioural ecology to go beyond adoption dynamics when studying the evolutionary process of cultural change.

## Research transparency and reproducibility

The data and R script for producing Figure 1 are available from https://figshare.com/s/d3492feab9aceddd2001
